# *Schistosoma mansoni* Presenting as a Zosteriform Papular Eruption

**DOI:** 10.4269/ajtmh.24-0743

**Published:** 2025-03-04

**Authors:** Arjun Chandna, Nilesh Morar, Nicholas Francis, Stephen L. Walker

**Affiliations:** ^1^ Hospital for Tropical Diseases, University College London Hospitals NHS Foundation Trust, London, United Kingdom;; ^2^ Cambodia Oxford Medical Research Unit, Angkor Hospital for Children, Siem Reap, Cambodia;; ^3^ Centre for Tropical Medicine & Global Health, University of Oxford, Oxford, United Kingdom;; ^4^ Department of Dermatology, Chelsea and Westminster Hospital NHS Foundation Trust, London, United Kingdom;; ^5^ Division of Histopathology, Cytology, and Immunology, Imperial College Hospitals NHS Trust, London, United Kingdom;; ^6^ Department of Dermatology, University College London Hospitals NHS Foundation Trust, London, United Kingdom;; ^7^ Faculty of Infectious & Tropical Diseases, London School of Hygiene and Tropical Medicine, London, United Kingdom

**Figure 1.**
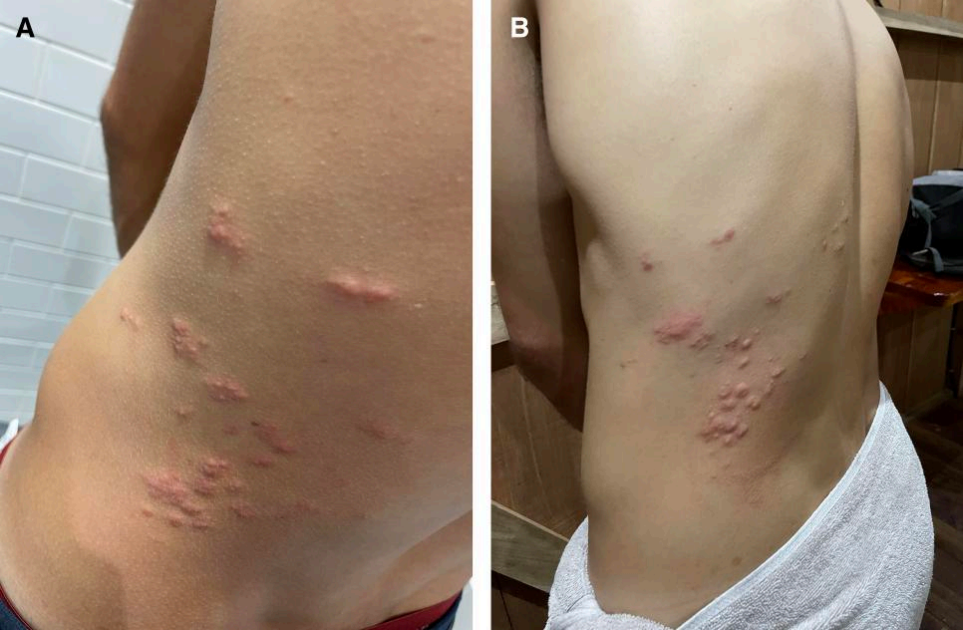
(**A**) Papular eruption in the T8–T10 dermatomes on the left side of the back at presentation. (**B**) Swelling of the papular eruption 6 weeks after initial presentation.

A 27-year-old man was seen for a pruritic rash on the lower back that had been present for 2 days. He had been troubled by diffuse, intermittent pruritus requiring antihistamines for the previous 6 months. He had no other medical history. On examination, firm erythematous papules were present on the left side of the back in the distribution of the T8–T10 dermatomes (Figure 1A). There was no lymphadenopathy. A clinical diagnosis of herpes zoster was made, and 7 days of oral acyclovir (400 mg five times per day) were prescribed. Blood tests showed mild leukocytopenia without eosinophilia (3.7 × 10^3^ cells/µL; reference range: 4.0–11.0 cells/µL) and elevated creatine kinase (460 IU/L; reference range: 40–320 IU/L). Total immunoglobulin E (IgE) was normal. Specific IgE testing was negative for a range of allergens.

The rash flared 6 weeks later, with intense pruritus and transient swelling of the papules (Figure 1B). Repeat blood tests indicated a mildly elevated alanine transaminase (ALT: 84 IU/L; reference range: <41 IU/L) and a marked increase in the creatine kinase (3,971 IU/L).

**Figure 2.**
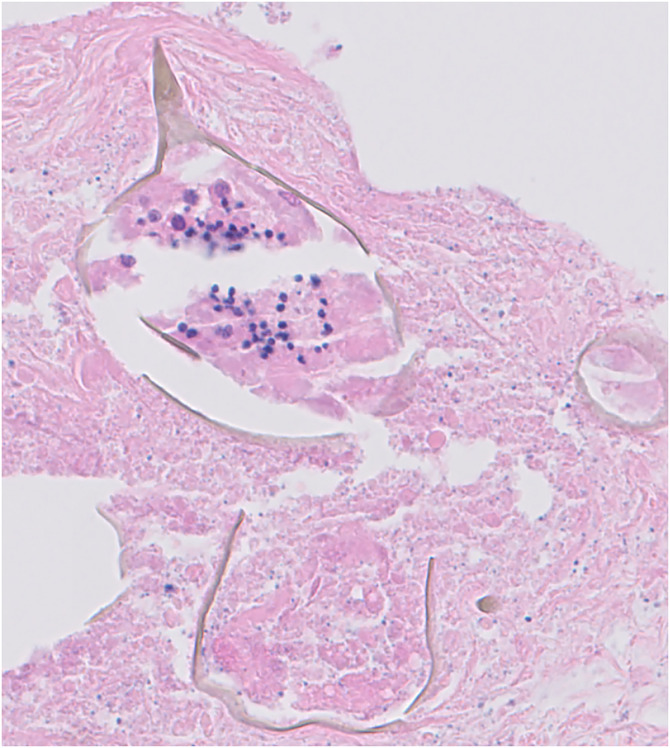
Hematoxylin and eosin stain of the skin punch biopsy demonstrating a schistosome ova with a well-preserved lateral spine.

Intermittent flares of the rash continued. Six weeks later, a skin biopsy showed dermal necrobiosis and a necrotizing granuloma surrounding a schistosome ovum with lateral spines, consistent with *Schistosoma mansoni* (Figure 2).

The patient was referred to our specialist infectious diseases center. On further questioning, he reported travel to Botswana, Mozambique, Uganda, Zambia, and Zimbabwe 6 years previously and swam in the Okavango delta, Lake Victoria, Lake Kariba, and the Zambezi. Examination of the abdomen was normal. Microscopic hematuria was absent, and no ova were visualized in stool or terminal urine samples. *Schistosoma* serology was strongly positive (1.18 optical density units; reference range: 0–0.26). HIV serology was negative. A single dose of oral praziquantel (40 mg/kg) was prescribed. Twelve weeks after praziquantel, he was still experiencing short-lived mild flares and was prescribed clobetasol propionate 0.05% ointment.

Schistosomiasis is caused by several trematode flukes of the genus *Schistosoma*.^1^ Cutaneous manifestations of schistosomiasis are urticaria and angioedema of Katayama syndrome—a hypersensitivity reaction characterized by cough, fever, and abdominal pain, typically occurring 2–8 weeks after infection. In chronic forms, the most common of which are firm, pruritic, papular, and verrucous lesions, the lower abdomen and genitals are often affected.^2^ A zosteriform or segmental presentation is rare and thought to relate to aberrant migration of ova to the spinal vessels through the vertebral plexus, resulting in this localized distribution. Zosteriform lesions typically appear within 6 months of infection. This patient shows that the interval may be longer, and a lifetime exposure history should be undertaken. A skin biopsy should be performed in individuals with nonresolving dermatomal eruptions as inflammatory and neoplastic conditions may be responsible.

Cutaneous schistosomiasis in this case reflects aberrant migration of *S. mansoni* eggs, and biopsy was required for diagnosis. Serological testing can be helpful, especially in travelers, but does not differentiate active infection from past exposure for individuals who live in endemic settings. Treatment with praziquantel is well tolerated and can be delivered in a single dose, but it is only active against mature adult *Schistosoma*; therefore, must be repeated 3–6 weeks later in individuals exposed in the preceding 3 months.
